# Validation of the Lithuanian version of the Brief Negative Symptoms Scale

**DOI:** 10.1192/j.eurpsy.2025.2239

**Published:** 2025-08-26

**Authors:** J. Montvidas, E. Zauka, V. Adomaitienė

**Affiliations:** 1 Psychiatry, Lithuanian University of Health Sciences, Kaunas, Lithuania

## Abstract

**Introduction:**

Negative symptoms of schizophrenia include abulia, anhedonia, alogia, blunted affect, and social isolation. These symptoms strongly correlate with health-related quality of life and treatment outcomes. (Azaiez et al., 2018; Galderisi et al., 2018; Kirkpatrick et al., 2006). According to current negative symptoms diagnosis and treatment guidelines, the Brief Negative Symptom Scale (BNSS) is the instrument of choice for the psychometric evaluation of negative symptoms (Galderisi et al., 2021). Unfortunately, BNSS was not available in Lithuania.

**Objectives:**

To validate the Lithuanian version of the BNSS in a Lithuanian-speaking sample.

**Methods:**

We performed a double translation from English to Lithuanian and then back to English. The final version of the Lithuanian BNSS (Lit-BNSS) was finalized according to comments from two native Lithuanian-speaking experts, who evaluated the forward translation, and the representatives of the authors of the BNSS, who evaluated the back translation. We performed a validation study in an inpatient setting in a university hospital in Lithuania and asked patients diagnosed with schizophrenia spectrum diagnosis according to ICD-10 to participate in the study. We evaluated the included patients with the Positive and Negative Symptoms Scale (PANSS), Montgomery Asberg Depression Rating Scale (MADRS), Self-Evaluation of Negative Symptoms Scale (SNS), and Calgary Depression Scale for Schizophrenia (CDSS). PANSS Marder factors were calculated for more accurate PANSS scores. We check the convergent validity with the Marder negative symptoms factor, the total score of SNS, and the discriminant validity with the Marder positive symptoms factor, MADRS, and CDSS total scores.

**Results:**

The study included 122 patients. The Lit-BNSS showed great internal consistency for the 13 items (α=0,944) and good consistency for six subscores (α=0,874). Convergent validity was good, with the total score of Lit-BNSS having a strong positive correlation with the Marder negative symptoms factor and a weaker correlation with the SNS total score. Discriminant validity was adequate because there were insignificant correlations with MADRS and CDSS subscores and the Marder positive symptoms factor. Correlation scores can be seen in Table 1.

**Table 1.** BNSS TS correlation with other scores. MARDER-NEG – PANSS Marder negative symptoms factor; SNS-TS- SNS total score; MARDER-POZ- PANSS Marder positive symptoms factor; MADRS – MADRS total score; CDSS-TS- CDSS total score

Variable	Correlation coefficient	p-value
MARDER-NEG	0,755	<0,001
SNS-TS	0,304	0,001
MARDER-POZ	0,171	0,064
MADRS-TS	0,085	0,361
CDSS-TS	0,472	0,117

**Conclusions:**

The Lit-BNSS is a valid and effective psychometric tool for evaluating negative symptoms in a Lithuanian-speaking sample.

**Disclosure of Interest:**

None Declared

